# The music-related quality of life: Italian validation of MuRQoL into MUSQUAV questionnaire and preliminary data from a cohort of postlingually deafened cochlear implant users

**DOI:** 10.1007/s00405-022-07258-1

**Published:** 2022-01-28

**Authors:** A. Frosolini, D. Parrino, A. Mancuso, N. Coppola, E. Genovese, C. de Filippis

**Affiliations:** 1 Department of Neuroscience DNS, University of Padova, Audiology Unit at Treviso Hospital, Piazzale Ospedale 1, 31100 Treviso, Italy; 2 Department of Information Science, University of Milan, Milan, Italy; 3 Audiology, Department of Diagnostic, Clinical and Public Health Medicine, University of Modena and Reggio Emilia, Modena, Italy

**Keywords:** Music, Cochlear implant, Music therapy, Hearing loss, Questionnaire

## Abstract

**Purpose:**

Cochlear implant (CI) users do not receive much of the auditory information necessary for an accurate perception of music. This usually entails a dissatisfaction with the music they hear, so that their quality of life may potentially be affected. The main aim of this paper was to translate and validate into Italian an instrument to evaluate these aspects—The Music-Related Quality of Life Questionnaire (MuRQoL)—to help the work of clinicians and therapists.

**Methods:**

The translation of the MuRQoL into “Questionario Musica e Qualità della Vita” (MUSQUAV) was done according to the international guidelines. The translated questionnaire was administered to normal hearing (NH) and CI users adults. Exploratory factor analysis, confirmatory factor analysis and known group method were used to confirm construct validity and applicability of MUSQUAV.

**Results:**

We retrieved 225 results. The MUSQUAV questionnaire was acceptable according to the goodness-of-fit indices. The correlation between the items, evaluated using Cronbach’s α coefficient, indicates a good internal consistency (> 0.80). The non-parametric Mann–Whitney test showed significant differences in the distinct populations tested.

**Conclusions:**

The MUSQUAV questionnaire is a valid, low-cost and rapid instrument for professional workers in the audiological field, especially useful in the assessment of the patients' perception and musical engagement.

**Supplementary Information:**

The online version contains supplementary material available at 10.1007/s00405-022-07258-1.

## Introduction

### Strengths and limits of electric hearing

Cochlear implants (CI) have definitely revolutionized the quality of life of people with profound hearing loss by restoring hearing functionality, improving their communication skills and social life [1].

Most CI users can benefit from a very good speech perception as their scores in pure tone audiometry and word recognition tests can result, in the best-case scenario, close to those of people with normal hearing. On the other hand, CI users do not spontaneously receive much of the temporal fine structure cues necessary for an accurate perception of pitch and timbre in music [2]. This usually entails dissatisfaction of the music they hear. This issue may potentially affect CI users’ individual quality of life, considering the pervasive presence of music in daily life and its role in emotional expression, social and cultural connection [3].

### Relevance of music perception in CI users

Emerging music-focused CI technologies [4, 5] and auditory music training programs may improve several aspects of music perception and enjoyment [6]. For these reasons, evidence about the efficacy of music focused interventions are needed to enable patients and clinicians to make informed decisions about how much time and money they should invest.

A recent systematic review concluded that no single test has been adopted into widespread use, in a research or clinical context, to assess music experience after cochlear implantation [7]. Music outcomes in CI users have been measured with objective tests such as the MuSIC test, the Montreal battery of the evaluation of amusia, the Clinical Assessment of Music Perception and other instruments. However, they still are scarcely used, since the application of music perception tests requires specific instrumentation, professional expertise and more time than conventional audiometric tests. Above all, their scores do not necessarily correlate with patients' music appraisal, enjoyment, and participation in musical activities [8]. Consequently, questionnaires are relevant for evaluating the music perception and engagement in CI users.

With this aim, many questionnaires have been validated: the Munich Music Questionnaire (MUMU) [9], the University of Canterbury Music Listening Questionnaire (UCMLQ) [10], the Iowa Musical Background Questionnaire (IMBQ) [8] and the Music Related Quality of Life Questionnaire (MuRQoL) [3].

### Need for an Italian version of the music-related self-report assessment

To the best of our knowledge, no Italian versions of subjective music assessment tools are available to date. In our opinion, among the above-mentioned questionnaires, the MuRQoL presents several advantages: (i) it is structured on a 5-point Likert scale, (ii) it involves both music perception and music engagement areas, (iii) it can be plotted in a matrix allowing the identification of specific areas to be improved through tailored musical training programs, and (iv) it is quite rapid to perform. A Turkish version has recently been validated as a helpful tool in the evaluation and rehabilitation of advanced sound perception in patients with electric hearing [11].

The main aim of this paper was to translate and validate an Italian version of the MuRQoL, to make it broadly available to clinicians and therapists. Two cohorts of normal hearing (NH) adults were firstly administered the translated questionnaire: one of musicians/music students and the other one of people not necessarily involved in musical practices. Additionally, we evaluated the preliminary results from its application in a group of CI users, to direct future research in the field.

## Material and methods

### Translation of the instrument

The translation of the “Music related quality of life questionnaire” (MuRQoL) into an Italian version “Questionario Musica e Qualità della Vita” (MUSQUAV) was done according to the recent international guidelines of translation and cross-cultural validation of healthcare scales [12]. The questionnaire is made up of 2 mirror sections, each one containing 18 questions. The first section, called “frequency”, analyzes how often the subject is able to perceive and be engaged in music whereas the second section, called “importance”, examines how important it is for the subject to perceive and be engaged in music [3].

Firstly, two native Italian speakers, bilingual in English, independently translated the original questionnaire into Italian, with the permission of the author. In the second phase, a comparison was made of the two translated versions of the instrument and a synthesis was achieved with the consensus of the entire study group. In the third phase, a professional translator translated the obtained pooled version into English. The original questionnaire and back translation were compared with each other for coherence, and the initial MusQuaV version was then formulated for patient testing. The final phase of the translation process, the patient testing panel, was attended by five medical doctors and five CI users, patients of the audiological tertiary centers of the University Hospitals of Treviso. The participants (six female, four male) were native speakers of the Italian language. Every item of the initial Italian version was read aloud; at the same time, participants could follow the text on printed copies of the instrument. During the group discussion the patients were invited to answer two questions: “What does this statement mean to you?” and “Is there any other wording that would enable this meaning to be expressed more clearly?”. Subsequent processing by the authors took the position of the testing panel into account and resulted in the elaboration of the Italian version of the MuRQoL, the MUSQUAV (Online Resource n1).

### Participants

The study was conducted in accordance with the principles of the Helsinki Declaration [13]. Data were examined in compliance with Italian privacy and sensitive data laws, and with the in-house rules of our institution. Informed consent was obtained from each participant.

As previously reported, 160 subjects were necessary to perform cross-cultural validity study of a questionnaire consisting of 18 items [11]. Our normal hearing (NH) validation sample consisted of amateur musician university students and amateur sport practitioners. We also report the preliminary data obtained on a cohort of CI patients of our department. Inclusion criteria were the following: (i) post-lingual deafness; (ii) unilateral/bilateral CI at least 18 months before questionnaire compilation; (iii) regular follow-up controls at the audiological tertiary centers of the University Hospital of Treviso. Demographic and clinical data (age, gender, etiology of deafness, years of CI use, CI model and audiological outcome) were recorded and are summarized in Table 1.

**Table 1:** Demographic and general characteristics of participants

	Student of music-informatics	Amateur sport practitioners	CI patients
Number of participants	88	97	35 (44 CIs)
Average age (years)	33.10 ± 17.25	34.56 ± 10.49	60.31 ± 17.01
Sex: female; male; non-binary	34; 52; 2	61; 36	21; 14
Mean subjective evaluation of hearing*	8.95 ± 1.04	9.01 ± 1.48	6.23 ± 1.97
Musical studies	66/88 (75%)	27/97 (27.83%)	2/35 (5.71%)
Professional musician	15/88 (17.04%)	1/97 (1.03%)	0/35
Etiology of hearing loss	NA	NA	Unknown (16); MD (5); Otosclerosis (4); Chronic otitis (4); AIED (3); Meningitis (2)
Hearing rehabilitation modality	NA	NA	Unilateral (7 Right, 6 Left); Bimodal (13); Bilateral (9)
Average years of CI use**	NA	NA	6.25 ± 6.01
CI model	NA	NA	Medel (20); Cochlear (13); Oticon (9); AB (2)
PTA2 unaided: Right; Left (dB)	NA	NA	104.53 ± 24.97; 105 ± 24.24
PTA2 with CI: Right; Left (dB)	NA	NA	37 ± 14.04; 37.5 ± 16.09
50% SRT with CI: Right; Left (dB)	NA	NA	46.19 ± 12.71; 44.96 ± 9.49

*AIED* Autoimmune Inner Ear Disease, *CI* Cochlear Implant, *MD* Meniere’s Disease, *NA* Not Applicable, *PTA2* Pure Tone Average at 500–1000-2000-4000 Hz, *SRT* Speech Recognition Threshold

*In visual-analog scale from 0 to 10

**Considered from the date of first CI in bilateral users

### Data collection

The final version of MusQuaV was administered in most of the cases by directly inviting participants to fill in an online survey (Online Resource n1), and by manual compilation of the same survey for a subset of participants attending our clinic at Treviso Hospital. Data were collected from 18 May 2021 to 18 August 2021. Open questions regarding subjective hearing evaluation, precedent hearing pathologies and musical background were included in the survey and taken into account.

For the CI users group, audiometric test results at last evaluation (within 6 months before questionnaire submission) were considered. Audiometry was performed with Madsen Astera by GN Otometrics (Denmark), in accordance with european (IEC 60645-I) and ISO (389-1) standards, in a sound-attenuating room. We tested hearing thresholds without and with hearing devices, in the best aided condition, at frequencies from 250 to 8000 Hz and the mean Pure Tone Average (PTA2, threshold levels at 0.5, 1, 2, and 4 kHz) was then calculated. For speech audiometry, articulation gain curves were obtained using disyllabic, phonetically balanced words from an Italian wordlist for adults [Bocca]. The Speech Reception Threshold (SRT)—decibel level at which 50% of words could be repeated by the subject—was considered.

### Validity and reliability of the MUSQUAV questionnaire

Only surveys filled out completely were taken into account for our analysis. Firstly, exploratory factor analysis (EFA) was performed for 18 frequency and 18 importance items separately, to find a factor based on the relationships between the variables. Principal axis factoring extraction method and 'oblimin' rotation were used [14]. Kaiser–Meyer–Olkin (KMO) and Bartlett’s test of sphericity were used to test the significance of factor structures. A KMO value close to one indicates sampling adequacy whereas the item must be removed if a factor load is below 0.30; a Bartlett test value p < 0.05 confirms sampling adequacy [15].

The construct validity of the questionnaire was measured through Confirmatory Factor Analysis (CFA) and the ‘known group’ method. Independent evaluation criteria, known as goodness-of-fit indices, were used to evaluate the results of the CFA. The fit of the model was determined using the root mean square error of approximation (RMSEA), as previously reported [11].

It was aimed to use the known-group method to evaluate the construct validity of the questionnaire and its ability to detect differences by comparing the scores of normal hearing music players, normal hearing sport players and post-lingual deaf CI users. It can be stated that a scale is valid if it gives significantly different scores for groups that are known to be apart from one another in a specific concept. The non-parametric Mann–Whitney test was used to verify this hypothesis, a *p* value < 0.05 was considered significant.

The open source software jamovi version 1.6, the SPSS Version 25.0 and R core team version 4.0 were used for our statistical purposes [16, 17].

## Results

Our normal hearing (NH) validation sample consisted of 88 amateur musician university students with an average age of 33.10 ± 17.25 years and 97 amateur sport practitioners with an average age of 34.56 ± 10.49 years. Other demographic and general data are summarized in Table 1.

The MUSQUAV questionnaire was acceptable according to the goodness-of-fit indices obtained as a result of the CFA for both scales. As shown in Table 2, root mean square error of approximation (RMSEA) and standardized root mean square residual (SRMR) resulted within normal limits: SRMR 0.0758 for Frequency and SRMR 0.0683 for Importance(0.05 ≤ SRMR ≤ 0.10 indicate acceptable fit) and RMSEA 0.0842 for Frequency and 0.104 for importance (0.05 ≤ RMSEA ≤ 0.10 indicate acceptable fit) [18].

**Table 2:** CFA Goodness-of-fit indices of the Italian version of the music-related quality of life questionnaire (*N* = 171)

			Italian	
Fit indices	Good fit	Acceptable fit	Frequency scale	Importance scale
*N*	—	—	171	171
df	—	—	134	134
*χ2*	0 ≤ χ2 ≤ 2 df	2 df ≤ χ2 ≤ 3 df	296	384
p	0.05 ≤ *P* ≤ 1.00	0.001 ≤ *P* ≤ 0.05	< 0.001	< 0.001
*χ2*/df	0 ≤ χ2/df ≤ 2	2 ≤ χ2/df ≤ 3	2.209	2.866
SRMR	0.00 ≤ SRMR ≤ 0.05	0.05 ≤ SRMR ≤ 0.10	0.0758	0.0683
RMSEA	0.00 ≤ RMSEA ≤ 0.05	0.05 ≤ RMSEA ≤ 0.10	0.0842	0.104
CFI	0.95 ≤ CFI ≤ 1.00	0.90 ≤ CFI ≤ 0.95	0.83	0.848
TLI	0.95 ≤ TLI ≤ 1.00	0.90 ≤ TLI ≤ 0.95	0.806	0.827

*CFI* comparative fit index, *RMSEA* root mean square error of approximation, *SRMR* standardized root mean square residual, *TLI* Tucker–Lewis index

**Fig. 1:**
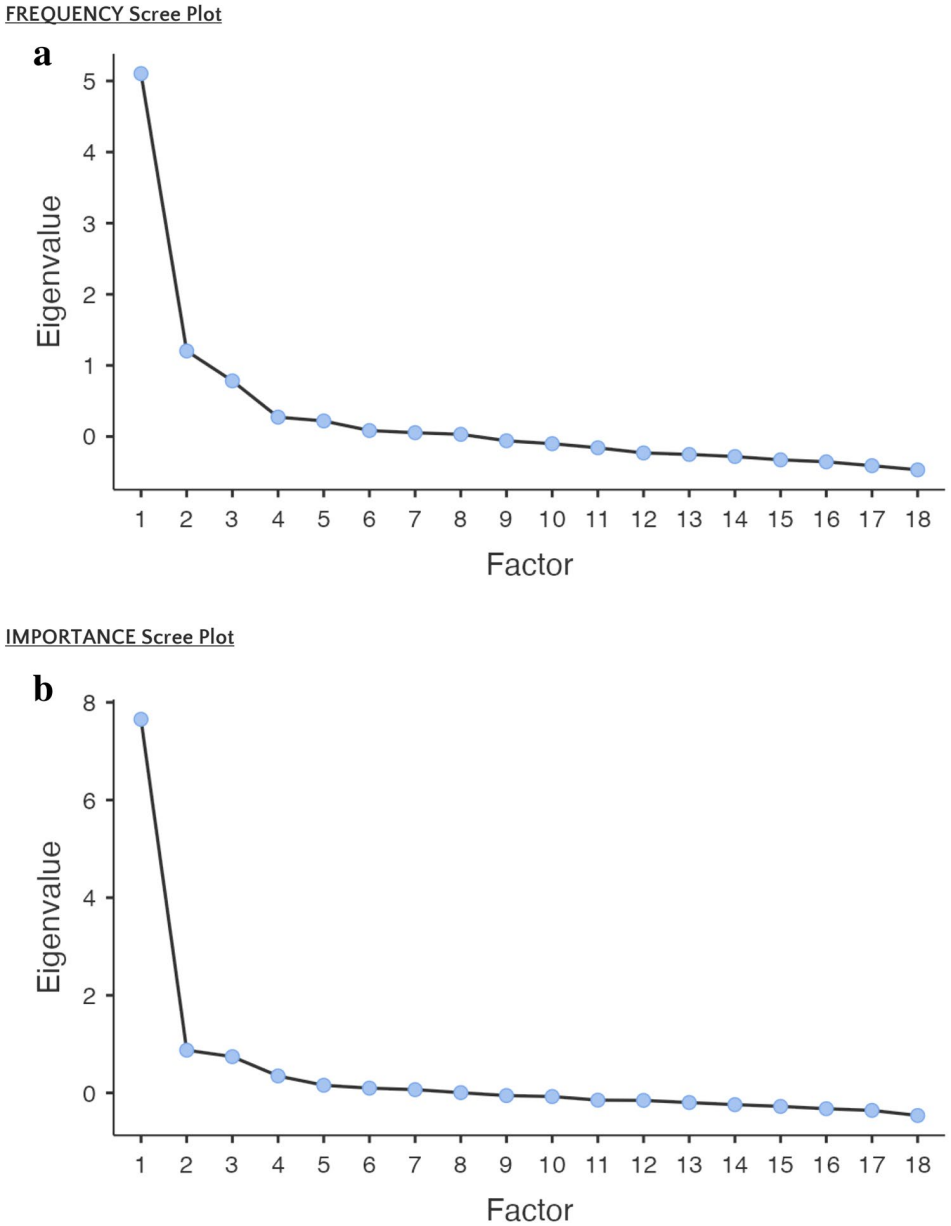
**a**, **b** Scree plots of exploratory factor analysis on the ‘Frequency scale’ and on the ‘Importance scale’ of MUSQUAV

Two factors (called factor 1 and factor 2) for ‘Frequency Scale’ and for ‘Importance Scale’ were extracted based on the first two eigenvalues and scree plots, as shown in Fig. 1a and b. As in the original questionnaire, factor 1 was interpreted as ‘music perception’ and factor 2 as ‘music engagement’ in both scales. Results obtained from EFA are presented in Table 3 third, fourth and fifth columns). As each item on a factor had a loading, it was determined that the 18 items in the MUSQUAV-frequency scale and 18 in the MUSQUAV-importance scale provided structural validity. In the case of importance, we preferred to keep questions 17 and 18 in the involvement factor to maintain consistency between the 2 groups of 18 questions. The dimensions obtained were structurally significant according to the results of the Bartlett test (by frequency scale: X2 = 1058, *p* < 0.001; by scale of importance: X2 = 1714, p < 0.001). The *p* value < 0.005 confirms that the variances of the two factors (perception and engagement) are different.

**Table 3:** KMO measure of sampling adequacy and exploratory factor analysis for the two sessions of the MUSQUAV questionnaire

Frequency session of the MUSQUAV	KMO MSA	Factor 1	Factor 2	Uniqueness
Overall	0.859	NA	NA	NA
1. Riesci a distinguere diversi ritmi musicali?	0.902	0.571	–	0.527
2. Riesci a seguire una melodia (ad esempio la melodia di una canzone o di un motivo familiare)?	0.894	0.662	–	0.406
3. Riesci a sentire le differenze di dinamica (cioè se la musica è ad alto o a basso volume)?	0.899	0.715	–	0.528
4. Riesci a riconoscere le parole nelle canzoni?	0.831	0.595	–	0.699
5. Riesci a distinguere il suono dei diversi strumenti musicali (violino, pianoforte, sassofono, chitarra…)?	0.912	0.565	–	0.554
6. Riesci a percepire il significato della musica (cioè l'emozione, perché è stata creata, quale messaggio vuole comunicare)	0.888	0.404	–	0.701
7. Riesci a sentire la musica senza bisogno di sforzarti, senza doverti concentrare?	0.882	0.770	–	0.434
8. Riesci a riconoscere una musica che ti è familiare (ad esempio una canzone, un cantante o una melodia)?	0.897	0.664	–	0.577
9. Sai giudicare la qualità di una performance musicale (ad esempio il cantato o la parte strumentale)?	0.813	0.423	–	0.660
10. Pensi di udire la musica come tutti gli altri?	0.559	0.387	0.349	0.847
11. Percepisci come intonata la musica che ascolti?	0.889	0.625	–	0.647
12. Ti piace la musica in ambienti rumorosi (ad esempio ad una festa, al ristorante o in auto) in assenza di stimoli visivi?	0.730	–	0.459	0.817
13. Ti piace ascoltare la musica in TV, DVD, smartphone o sul computer quando è possibile seguire la performance anche visivamente?	0.774	–	0.579	0.677
14. Metti la musica in sottofondo mentre fai qualcos'altro (ad es. durante la lettura, la pittura, il giardinaggio, i lavori domestici, l'esercizio o semplicemente il relax)?	0.779	–	0.423	0.798
15. Ascolti musica mentre viaggi (ad esempio in auto)?	0.781	–	0.302	0.771
16. Ascolti musica nuova, che non hai mai sentito prima?	0.854	–	0.540	0.588
17. Partecipi a eventi musicali (ad esempio musical, concerti o festival musicali)?	0.842	–	0.546	0.717
18. Canti, suoni uno strumento musicale o fischietti quando sei da solo?	0.916	–	0.606	0.568

Importance session of the MUSQUAV	KMO MSA	Factor 1	Factor 2	Uniqueness
Overall	0.908	NA	NA	NA
1. Quanto è importante per te riuscire a distinguere diversi ritmi musicali?	0.906	0.836	–	0.318
2. Quanto è importante per te riuscire a seguire una melodia (ad esempio la melodia di una canzone o di un motivo familiare)?	0.916	0.859	–	0.289
3. Quanto è importante per te riuscire a sentire le differenze di dinamica (cioè se la musica è ad alto o a basso volume)?	0.943	0.783	–	0.405
4. Quanto è importante per te riuscire a riconoscere le parole nelle canzoni?	0.874	0.595	–	0.627
5. Quanto è importante per te distinguere il suono dei diversi strumenti musicali (violino, pianoforte, sassofono, chitarra…)?	0.933	0.861	–	0.341
6. Quanto è importante per te riuscire a percepire il significato della musica (cioè l'emozione, perché è stata creata, quale messaggio vuole comunicare)	0.900	0.577	–	0.565
7. Quanto è importante per te riuscire a sentire la musica senza bisogno di sforzarti, senza doverti concentrare?	0.897	0.562	–	0.603
8. Quanto è importante per te riuscire a riconoscere una musica che ti è familiare (ad esempio una canzone, un cantante o una melodia)?	0.911	0.614	–	0.553
9. Quanto è importante per te riuscire a giudicare la qualità di una performance musicale (ad esempio il cantato o la parte strumentale)?	0.934	0.778	–	0.421
10. Quanto è importante per te la consapevolezza di udire la musica come tutti gli altri?	0.837	0.397	–	0.808
11. Quanto è importante per te percepire come intonata la musica che ascolti (armonica, melodiosa)?	0.936	0.676	–	0.545
12. Quanto è importante per te apprezzare la musica in ambienti rumorosi (ad esempio ad una festa, al ristorante o in macchina) in assenza di stimoli visivi?	0.911	0.381	0.319	0.636
13. Quanto è importante per te ascoltare musica su TV, DVD, smartphone o sul computer quando è possibile seguire la performance anche visivamente?	0.905	0.366	0.411	0.551
14. Quanto è importante per te avere musica in sottofondo mentre fai qualcos'altro (ad esempio durante la lettura, la pittura, il giardinaggio, i lavori domestici, l'esercizio o semplicemente il relax)?	0.806	–	0.811	0.403
15. Quanto è importante per te ascoltare musica mentre viaggi (ad esempio in auto)?	0.844	–	0.717	0.435
16. Quanto è importante per te ascoltare musica nuova, che non hai mai sentito prima?	0.907	0.353	0.401	0.577
17. Quanto è importante per te partecipare a eventi musicali (ad esempio musical, concerti o festival musicali)?	0.918	0.418	0.283	0.630
18. Quanto è importante per te cantare, suonare uno strumento musicale o fischiettare quando sei da solo?	0.944	0.526	0.286	0.497

*KMO* Kaiser–Meyer–Olkin, *MSA* Measure of Sample Adequacy, *NA* Not Applicable

'Principal axis factoring' extraction method was used in combination with a 'oblimin' rotation. Factor 1 corresponds to “Percezione” (Perception) and Factor 2 correspond to “Coinvolgimento” (Engagement) (Dritsakis et al. [3])

The two factors chosen in the exploratory analysis account for 36% cumulative frequency of the variance (factor 1 23.1% and factor 2 12.9%) and for importance cumulatively 48.9% (factor 1 36, 7% and factor 2 12.1%).

Table 2 shows the CFA values compared with the reference values.

The correlation between the items of the questionnaire evaluated using Cronbach’s α coefficient, reported 0.848 for frequency scale and 0.925 for importance scale, indicating a good internal consistency for both scales. (> 0.80), The estimated Cronbach’s α coefficients for correlation between the items of each subscale were 0.851 (music perception subscale) and 0.737 (music engagement subscale) (0.6 < α < 0.8 indicate acceptable validity) for frequency scale and 0.911 (music perception subscale) and 0.838 (music engagement subscale) for importance scale.

The cohort of CI patients consisted of 20 females and 15 males with an average age of 60.31 years (standard deviation ± 17.01). The non parametric Mann–Whitney test showed significant differences in the results of importance and frequency scales of MUSQUAV in the three populations tested. These data, summarized in Table 4 further confirmed the reliability of the questionnaire in identifying differences based on musical experience of participants, with higher values of frequency for the normal hearing group compared with the cochlear implant group and similar values of importance in the different groups, as previously reported [3].

**Table 4:** The non-parametric Mann–Whitney test showed significant differences in the results of importance and frequency scales of MUSQUAV in the three populations tested: NH Amateur Musicians vs NH Sport practitioner; NH vs CI, NH Amateur Musicians vs CI, NH Amateur Musician vs NH Sport practitioner

	CI		NH		Mann–Whithney	
Measure	Median	Range	Median	Range	*U*	*P*
Frequency	2.42	1.50–4.44	4.17	1.44–5.00	534	< 0.001
Importance	3.28	1.67–4.94	3.89	1.06–5.00	1516.5	0.002
Frequency Perception	2.86	1.64–4.73	4.36	1.18–5.00	556.5	< 0.001
Importance Perception	3.36	1.73–4.91	4.00	1.09–5.00	1639.5	0.008
Frequency Engagement	2.14	1.00–4.14	3.71	1.86–5.00	650.5	< 0.001
Importance Engagement	3.00	1.14–5.00	3.71	1.00–5.00	1490	0.001

	CI		NH-Mus		Mann–Whithney	
Measure	Median	Range	Median	Range	*U*	*P*
Frequency	2.42	1.50–4.44	4.28	1.44–5.00	217	< 0.001
Importance	3.28	1.67–4.94	4.00	2.50–5.00	518	< 0.001
Frequency Perception	2.86	1.64–4.73	4.36	1.18–5.00	250.5	< 0.001
Importance Perception	3.36	1.73–4.91	4.00	2.45–5.00	620	< 0.001
Frequency Engagement	2.14	1.00–4.14	4.14	1.86–5.00	234.5	< 0.001
Importance Engagement	3.00	1.14–5.00	4.00	1.86–5.00	516.5	< 0.001

	CI		NH-Sport		Mann–Whithney	
Measure	Median	Range	Median	Range	*U*	*P*
Frequency	2.42	1.50–4.44	3.94	2.61–4.83	317	< 0.001
Importance	3.28	1.67–4.94	3.58	1.06–5.00	998.5	0.132
Frequency Perception	2.86	1.64–4.73	4.23	2.36–5.00	306	< 0.001
Importance Perception	3.36	1.73–4.91	3.64	1.09–5.00	1019.5	0.17
Frequency Engagement	2.14	1.00–4.14	3.57	2.71–4.71	416	< 0.001
Importance Engagement	3.00	1.14–5.00	3.21	1.00–5.00	973	0.095

	NH-Mus		NH-Sport		Mann–Whithney	
Measure	Median	Range	Median	Range	*U*	*P*
Frequency	4.28	1.44–5.00	3.94	2.61–4.83	2390	< 0.001
Importance	4.00	2.50–5.00	3.58	1.06–5.00	2235.5	< 0.001
Frequency Perception	4.36	1.18–5.00	4.23	2.36–5.00	3048.5	0.0614
Importance Perception	4.00	2.45–5.00	3.64	1.09–5.00	2518	< 0.001
Frequency Engagement	4.14	1.86–5.00	3.57	2.71–4.71	2291.5	< 0.001
Importance Engagement	4.00	1.86–5.00	3.21	1.00–5.00	2210	< 0.001

*CI* Cochlear Implant, *NH* Normal Hearing group, *Mus* Amateur Musician

Multiple regressions were performed for the NH subjects (Table 5). As regards the importance, being a musician and having done musical studies are significant: the average value of importance decreases by 0.4 passing from musicians to non-musicians and increases by 0.4 with musical studies. Also for frequency, passing from musician to non-musician is significant (the average of the frequencies decreases by 0.17), and also the average decreases with the increasing age of participants (0.23 for each year).

**Table 5:** Results of multiple regression for the normal hearing group of participants

Frequency			
	Unstandardized coefficient	Standardized coefficient	*p*
Model	*B*	Beta	
(Constant)	3.972	—	< 0.05
Group (amateur musician—sport practitioners)	− 0.423	− 0.3	< 0.05
Gender	0.135	0.095	0.222
Age	− 0.005	− 0.092	0.232
Subjective hearing evaluation	0.039	0.014	0.847
Musical studies	0.21	0.149	0.071
Professional musicians	− 0.003	− 0.001	0.987

Importance			
	Unstandardized coefficient	Standardized coefficient	*p*
Model	*B*	Beta	
(Constant)	4.343	—	< 0.05
Group (amateur musician—sport practitioners)	− 0.163	− 0.178	0.029
Gender	0.085	0.092	0.235
Age	− 0.008	− 0.238	0.002
Subjective Hearing evaluation	0.09	0.05	0.491
Musical studies	0.113	0.123	0.133
Professional musicians	0.193	0.123	0.108

**Fig. 2:**
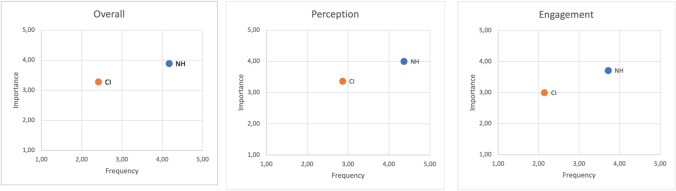
Matrix of frequency and importance for CI and NH: overall, perception and engagement

Figure 2 shows the combinations of frequency and importance scores following the classification proposed by Dristakis et al. [3]. This matrix visualization well synthesized the differences between the NH and CI populations. As can be seen the CIs are positioned in the top-left quadrant, with great importance and low frequency, and therefore with a highly critical impact from the perspective of rehabilitation.

## Discussion

Perception of music is a complex auditory and cognitive activity that can be especially challenging for patients with hearing loss [2]. The consequence of poor music engagement and participation can affect patients' Quality of Life (QoL). To the best of our knowledge, no evaluation instruments are available to assess the musical attitude of Italian patients with hearing loss [7]. This evidence has led us to the translation and validation of MuRQoL into this Italian version, the MUSQUAV.

The MurQoL was developed in 2017 by Dritsakis et al. with the contributions of a wide and qualified group of researchers in the field of audiology and with the direct participation of CI patients. The final version was validated on a cohort of 147 CI users and 68 normal hearing matched controls. The study resulted with high reliability of the questionnaire, for the test–retest reliability and the value of Cronbach's α that exceeded 0.90. Moreover, the ability of the MuRQoL questionnaire to predict aspects of QoL was shown by the positive correlation between MuRQoL frequency engagement and the SF12v2-RP domain, which covers activity limitations as a result of physical health. A subsequent validation in the Turkish language, on 161 CI users and 162 normal hearing controls, confirmed previous findings regarding reliability and validity of the questionnaire [11].

Our findings on 180 normal hearing controls and 35 CI patients are in line with the data previously reported by Dritsakis and Akbulut. This indicates cross-cultural validity of the MuRQoL questionnaire and validity and reliability of the Italian MUSQUAV. Moreover, our preliminary findings on CIs showed a great need for musical interventions for these patients (Fig. 2) and consequent possible impact on their quality of life.

The MUSQUAV questionnaire can be used as a screening tool to identify individual rehabilitation needs in a clinic, using the matrix diagram (available in the Online Resource n2). At a population level, the MUSQUAV can be used as a reliable and valid outcome measure for the evaluation of music-focused interventions on hearing impaired patients. A decision tree and score calculator, to aid researchers and clinicians in the use of the MUSQUAV, is available in the Online Resource n3 [3].

Future longitudinal studies should assess the ability of the MUSQUAV to detect clinical changes in patients who underwent music focused intervention, including possible correlations with language and communication abilities, which have recently been debated [19, 20]. The extension of music evaluation (including MUSQUAV questionnaire) and music focused interventions on people with mild hearing loss should also be considered, given the fact that hearing aids (HA) and CI share similar technologies—implication of bimodal hearing—and patients often pass from hearing aids to CI during their lifetime due to the natural course of hearing loss diseases.

In conclusion, the present MUSQUAV questionnaire—Italian version of MuRQoL—is a valid, low-cost and rapid instrument available for professional workers in the audiology field to assess perception and musical engagement of patients.

### Supplementary Information

Below is the link to the electronic supplementary material.

Supplementary file1 (PDF 178 KB)

## Data Availability

The data that support the findings of this study are available from the corresponding author (AF), upon reasonable request.
