# Correction to: The music‐related quality of life: Italian validation of MuRQoL into MUSQUAV questionnaire and preliminary data from a cohort of postlingually deafened cochlear implant users

**DOI:** 10.1007/s00405-023-08434-7

**Published:** 2024-01-11

**Authors:** A. Frosolini, D. Parrino, A. Mancuso, N. Coppola, E. Genovese, C. de Filippis

**Affiliations:** 1 Department of Neuroscience DNS, University of Padova, Audiology Unit at Treviso Hospital, Piazzale Ospedale 1, 31100 Treviso, Italy; 2 Department of Information Science, University of Milan, Milan, Italy; 3 Audiology, Department of Diagnostic, Clinical and Public Health Medicine, University of Modena and Reggio Emilia, Modena, Italy

**Correction to: European Archives of Oto-Rhino-Laryngology (2022) 279:4769–4778** 10.1007/s00405-022-07258-1

Regrettably, item 3 of the Musica e Qualità della Vita (MUSQUAV)—‘differenze di dinamica (cioè se la musica è ad alto o a basso volume)’—failed to accurately convey the intended meaning of the corresponding item in the original Music Related Quality of Life (MuRQoL), which is ‘differences in musical tone (i.e., how high or low the music is)’. Consequently, we revised item 3 in both the frequency and importance sessions to ‘differenze nel tono musicale (cioè quanto è acuta o grave la musica)?’. This change aligns with the original intent of the questionnaire. Additionally, we conducted the Exploratory Factor Analysis afresh, incorporating this correctly translated item. The survey was conducted on the original study sample in October 2023; complete responses from 52 subjects were retrieved. The revised Table 3 and supplementary materials are herein reported. Since its Italian translation, the MUSQUAV questionnaire has also been referred to as MuRQoL-It in official congresses and professional contexts. To the best of our knowledge, both acronyms—MUSQUAV and MuRQoL-It—are valid and appropriate for referring to the Italian version of the MuRQoL questionnaire.

**Table 3:** KMO measure of sampling Adequacy and Exploratory Factor Analysis for the two sessions of the MUSQUAV questionnaire

Frequency session of the MUSQUAV (MuRQoL-It)	KMO MSA	Factor 1	Factor 2	Uniqueness
*Overall*	0.430	–		
1. Riesci a distinguere diversi ritmi musicali?	0.428	0.518	–	0.733
2. Riesci a seguire una melodia (ad esempio la melodia di una canzone o di un motivo familiare)?	0.612	0.720	–	0.485
3. Riesci a sentire le differenze nel tono musicale (cioè quanto è acuta o grave la musica)?	0.548	0.614	–	0.592
4. Riesci a riconoscere le parole nelle canzoni?	0.288	–	–	0.947
5. Riesci a distinguere il suono dei diversi strumenti musicali (violino, pianoforte, sassofono, chitarra…)?	0.325	0.602	–	0.635
6. Riesci a percepire il significato della musica (cioè l’emozione, perché è stata creata, quale messaggio vuole comunicare)	0.538	–	0.484	0.571
7. Riesci a sentire la musica senza bisogno di sforzarti, senza doverti concentrare?	0.383	0.486	–	0.762
8. Riesci a riconoscere una musica che ti è familiare (ad esempio una canzone, un cantante o una melodia)?	0.378	0.420	–	0.819
9. Sai giudicare la qualità di una performance musicale (ad esempio il cantato o la parte strumentale)?	0.707	0.363	–	0.817
10. Pensi di udire la musica come tutti gli altri?	0.373		–	0.988
11. Percepisci come intonata la musica che ascolti?	0.210	–	–	0.983
12. Ti piace la musica in ambienti rumorosi (ad esempio ad una festa, al ristorante o in auto) in assenza di stimoli visivi?	0.342	–	0.465	0.757
13. Ti piace ascoltare la musica in TV, DVD smartphone o sul computer quando è possibile seguire la performance anche visivamente?	0.415	–	0.358	0.862
14. Metti la musica in sottofondo mentre fai qualcos’altro (ad es. durante la lettura, la pittura, il giardinaggio, i lavori domestici, l’esercizio o semplicemente il relax)?	0.392	–	0.833	0.304
15. Ascolti musica mentre viaggi (ad esempio in auto)?	0.340	–	0.688	0.547
16. Ascolti musica nuova, che non hai mai sentito prima?	0.526	–	0.656	0.476
17. Partecipi a eventi musicali (ad esempio musical, concerti o festival musicali)?	0.569	–	0.521	0.500
18. Canti, suoni uno strumento musicale o fischietti quando sei da solo?	0.458	0.689	–	0.521

Importance session of the MUSQUAV (MuRQoL-It)	KMO MSA	Factor 1	Factor 2	Unique-ness
*Overall*	0.646	–		
1. Quanto è importante per te riuscire a distinguere diversi ritmi musicali?	0.826	0.729	–	0.422
2. Quanto è importante per te riuscire a seguire una melodia (ad esempio la melodia di una canzone o di un motivo familiare)?	0.669	0.627	–	0.561
3. Quanto è importante per te riuscire a sentire le differenze nel tono musicale (cioè quanto è acuta o grave la musica)?	0.624	0.857	–	0.285
4. Quanto è importante per te riuscire a riconoscere le parole nelle canzoni?	0.554	–	0.460	0.762
5. Quanto è importante per te distinguere il suono dei diversi strumenti musicali (violino, pianoforte, sassofono, chitarra…)?	0.544	0.672	–	0.538
6. Quanto è importante per te riuscire a percepire il significato della musica (cioè l’emozione, perché è stata creata, quale messaggio vuole comunicare)	0.590	–	0.590	0.543
7. Quanto è importante per te riuscire a sentire la musica senza bisogno di sforzarti, senza doverti concentrare?	0.760	–	0.485	0.604
8. Quanto è importante per te riuscire a riconoscere una musica che ti è familiare (ad esempio una canzone, un cantante o una melodia)?	0.599	–	0.453	0.637
9. Quanto è importante per te riuscire a giudicare la qualità di una performance musicale (ad esempio il cantato o la parte strumentale)?	0.686	0.558	–	0.613
10. Quanto è importante per te la consapevolezza di udire la musica come tutti gli altri?	0.540	0.304	–	0.797
11. Quanto è importante per te percepire come intonata la musica che ascolti (armonica, melodiosa)?	0.674	0.505	–	0.737
12. Quanto è importante per te apprezzare la musica in ambienti rumorosi (ad esempio ad una festa, al ristorante o in macchina) in assenza di stimoli visivi?	0.659	–	0.540	0.646
13. Quanto è importante per te ascoltare musica su TV, DVD, smartphone o sul computer quando è possibile seguire la performance anche visivamente?	0.584	–	0.354	0.800
14. Quanto è importante per te avere musica in sottofondo mentre fai qualcos’altro (ad esempio durante la lettura, la pittura, il giardinaggio, i lavori domestici, l’esercizio o semplicemente il relax)?	0.539	–	0.649	0.600
15. Quanto è importante per te ascoltare musica mentre viaggi (ad esempio in auto)?	0.501	–	0.695	0.528
16. Quanto è importante per te ascoltare musica nuova, che non hai mai sentito prima?	0.725	0.526	–	0.660
17. Quanto è importante per te partecipare a eventi musicali (ad esempio musical, concerti o festival musicali)?	0.697	–	0.405	0.696
18. Quanto è importante per te cantare, suonare uno strumento musicale o fischiettare quando sei da solo?	0.857	0.603	–	0.644

‘Principal axis factoring’ extraction method was used in combination with a ‘oblimin’ rotation. Factor 1 corresponds to “Percezione” (Perception) and Factor 2 corresponds to “Coinvolgimento” (Engagement) (Dritsakis et al. 2017)

*KMO* Kaiser–Meyer–Olkin, *MSA* measure of sample adequacy

### Supplementary Information

Below is the link to the electronic supplementary material.

Supplementary file1 (PDF 163 KB)

