# Supplementary material for: The music-related quality of life: Italian validation of MuRQoL into MUSQUAV questionnaire and preliminary data from a cohort of postlingually deafened cochlear implant users
Source: Eur Arch Otorhinolaryngol. 2022 Jan 28;279(10):4769–78. doi: 10.1007/s00405-022-07258-1 (PMC9474524; doi:10.1007/s00405-022-07258-1)
Supplement: Supplementary file 1 — Supplementary file1 (PDF 178 KB) [file 405_2022_7258_MOESM1_ESM.pdf]

APPENDICES of the original article "THE MUSIC-RELATED QUALITY OF LIFE: ITALIAN VALIDATION OF MURQOL INTO MUSQUAV QUESTIONNAIRE AND PRELIMINARY DATA FROM A COHORT OF POSTLINGUALLY DEAFENED COCHLEAR IMPLANT USERS", written by *Frosolini A.; Parrino D.; Mancuso A.; Coppola N.; Genovese E.; de Filippis C.; Published in European Archives of Oto-Rhino-Laryngology and Head & Neck (Springer) 2022.*

APPENDIX 1. Questionario Musica e Qualità della Vita (MUSQUAV).

Direct LINK to the Online version: https://forms.gle/CtH6AUrRAatbNtLG7

Questionario "Musica e qualità della vita" MUSQUAV

Validazione italiana del questionario "Music-related quality of life" MuRQoL di Dritsakis et al. (2017)

La prima sessione del questionario (denominata FREQUENZA) riguarda le tue capacità di percezione della musica e il tuo coinvolgimento in attività di ascolto e pratica musicale.
Per favore rispondi a tutte le domande barrando il riquadro corrispondente ad una delle 6 possibili risposte: 1) mai; 2) raramente; 3) di tanto in tanto; 4) spesso; 5) sempre; NS) non saprei

**MUSQUAV sessione FREQUENZA**						
**Percezione della musica**	1	2	3	4	5	NS
1. Riesci a distinguere diversi ritmi musicali?						
2. Riesci a seguire una melodia (ad esempio la melodia di una canzone o di un motivo familiare)?						
3. Riesci a sentire le differenze di dinamica (cioè se la musica è ad alto o a basso volume)?						
4. Riesci a riconoscere le parole nelle canzoni?						
5. Riesci a distinguere il suono dei diversi strumenti musicali (violino, pianoforte, sassofono, chitarra...)?						
6. Riesci a percepire il significato della musica (cioè l'emozione, perché è stata creata, quale messaggio vuole comunicare).						
7. Riesci a sentire la musica senza bisogno di sforzarti, senza doverti concentrare?						
8. Riesci a riconoscere una musica che ti è familiare (ad esempio una canzone, un cantante o una melodia)?						
9. Sai giudicare la qualità di una performance musicale (ad esempio il cantato o la parte strumentale)?						
10. Pensi di udire la musica come tutti gli altri?						
11. Percepisci come intonata la musica che ascolti?						
**Coinvolgimento musicale**	1	2	3	4	5	NS
12. Ti piace la musica in ambienti rumorosi (ad esempio ad una festa, al ristorante o in auto) in assenza di stimoli visivi?						
13. Ti piace ascoltare la musica in TV, DVD, smartphone o sul computer quando è possibile seguire la performance anche visivamente?						
14. Metti la musica in sottofondo mentre fai qualcos'altro (ad es. durante la lettura, la pittura, il giardinaggio, i lavori domestici, l'esercizio o semplicemente il relax)?						
15. Ascolti musica mentre viaggi (ad esempio in auto)?						
16. Ascolti musica nuova, che non hai mai sentito prima?						
17. Partecipi a eventi musicali (ad esempio musical, concerti o festival musicali)?						
18. Canti, suoni uno strumento musicale o fischietti quando sei da solo?						

La seconda sessione del questionario (denominata IMPORTANZA) è composta dalle stesse domande della prima parte, le risposte in questo caso riguardano quanto sono importanti per te le capacità di percezione della musica e il coinvolgimento in attività di ascolto e pratica musicale.
Per favore, rispondi a tutte le domande barrando il riquadro corrispondente ad una delle 6 possibili risposte: 1) irrilevante; 2) non molto importante; 3) rilevante; 4) molto importante; 5) estremamente importante; NS) non saprei.

**MUSQUAV sessione IMPORTANZA**						
**Percezione della musica**	1	2	3	4	5	NS
1. Quanto è importante per te riuscire a distinguere diversi ritmi musicali?						
2. Quanto è importante per te riuscire a seguire una melodia (ad esempio la melodia di una canzone o di un motivo familiare)?						
3. Quanto è importante per te riuscire a sentire le differenze di dinamica (cioè se la musica è ad alto o a basso volume)?						
4. Quanto è importante per te riuscire a riconoscere le parole nelle canzoni?						
5. Quanto è importante per te distinguere il suono dei diversi strumenti musicali (violino, pianoforte, sassofono, chitarra...)?						
6. Quanto è importante per te riuscire a percepire il significato della musica (cioè l'emozione, perché è stata creata, quale messaggio vuole comunicare).						
7. Quanto è importante per te riuscire a sentire la musica senza bisogno di sforzarti, senza doverti concentrare?						
8. Quanto è importante per te riuscire a riconoscere una musica che ti è familiare (ad esempio una canzone, un cantante o una melodia)?						
9. Quanto è importante per te riuscire a giudicare la qualità di una performance musicale (ad esempio il cantato o la parte strumentale)?						
10. Quanto è importante per te la consapevolezza di udire la musica come tutti gli altri?						
11. Quanto è importante per te percepire come intonata la musica che ascolti (armonica, melodiosa)?						
**Coinvolgimento musicale**	1	2	3	4	5	NS
12. Quanto è importante per te apprezzare la musica in ambienti rumorosi (ad esempio ad una festa, al ristorante o in macchina) in assenza di stimoli visivi?						
13. Quanto è importante per te ascoltare musica su TV, DVD, smartphone o sul computer quando è possibile seguire la performance anche visivamente?						
14. Quanto è importante per te avere musica in sottofondo mentre fai qualcos'altro (ad esempio durante la lettura, la pittura, il giardinaggio, i lavori domestici, l'esercizio o semplicemente il relax)?						
15. Quanto è importante per te ascoltare musica mentre viaggi (ad esempio in auto)?						
16. Quanto è importante per te ascoltare musica nuova, che non hai mai sentito prima?						
17. Quanto è importante per te partecipare a eventi musicali (ad esempio musical, concerti o festival musicali)?						
18. Quanto è importante per te cantare, suonare uno strumento musicale o fischiettare quando sei da solo?						

APPENDIX 2. Matrice per l’utilizzo del Questionario Musica e Qualità della Vita (MUSQUAV). L'area evidenziata in grigio rappresenta aspetti della percezione e del coinvolgimento musicale che sono considerati importanti

dal paziente ma scarsamente godibili. Gli elementi che ricadono in quest'area della matrice possono evidenziare aree di interesse, elementi di discussione, esigenze riabilitative, e possono aiutare a definire il profilo del paziente e formare un programma di intervento specifico.

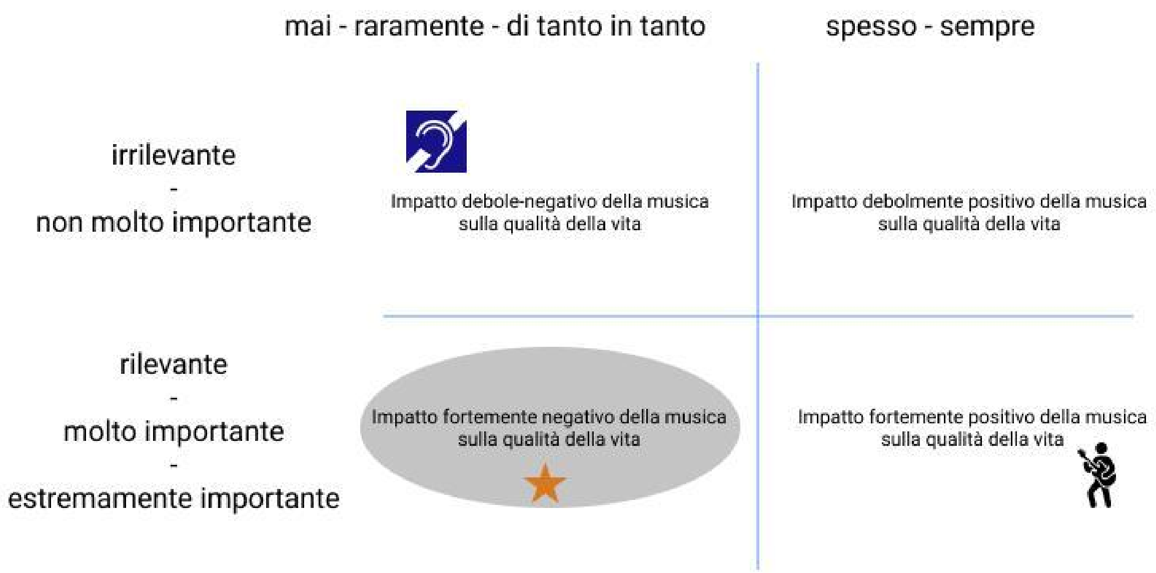

APPENDIX 3. Formule e diagramma per l'utilizzo del Questionario Musica e Qualità della Vita (MUSQUAV) e delle sue sub-scale nella valutazione degli outcome di interventi di riabilitazione della esperienza musicale, sia a livello individuale che a livello di studi di popolazione (Dritsakis et al. 2017).

**Come calcolare i punteggi medi**:
1. Converti i punteggi 1-5 per la PARTE I o per LA PARTE II in una scala 0-100 (1 = 0, 2 = 25, 3 = 50, 4 = 75, 5 = 100).
2. Sommare i punteggi per la PARTE I o LA PARTE II per ottenere le singole scale di PERCEZIONE MUSICALE e PARTECIPAZIONE MUSICALE oppure sommare tutti i punteggi per ottenere la scala TOTALE.
3. Dividere per il numero di elementi, ovvero 11 elementi solo per PERCEZIONE MUSICALE, 7 elementi per PERCEZIONE MUSICALE e 18 elementi per la scala OVERALL.
N.B.: Si raccomanda di scartare i questionari con > 3 N/S (Non Saprei) per una singola subscala.

**Come confrontare i punteggi prima e dopo l'intervento**:<br>sottrarre il punteggio medio prima dell'intervento dal punteggio medio dopo l'intervento. Se la differenza è

maggiore del corrispondente "più piccolo cambiamento rilevabile" (mostrato di seguito), è probabile che il risultato rifletta un vero cambiamento, non un cambiamento dovuto a errore casuale e/o di misurazione.
**Il più piccolo cambiamento rilevabile** (su scala 0-100):
● PARTE I (Frequenza) TOTALE (domande 1-18) = **10**
● PARTE I (Frequenza) PERCEZIONE MUSICALE (domande 1-11) = **15**
● PARTE I (Frequenza) COINVOLGIMENTO MUSICALE (domande 12-18) = **13**
● PARTE II (Importanza) TOTALE (domande 1-18) = **19**
● PARTE II (Importanza) PERCEZIONE MUSICALE (domande 1-11) = **23**
● PARTE II (Importanza) IMPEGNO MUSICALE (domande 12-18) = **23**

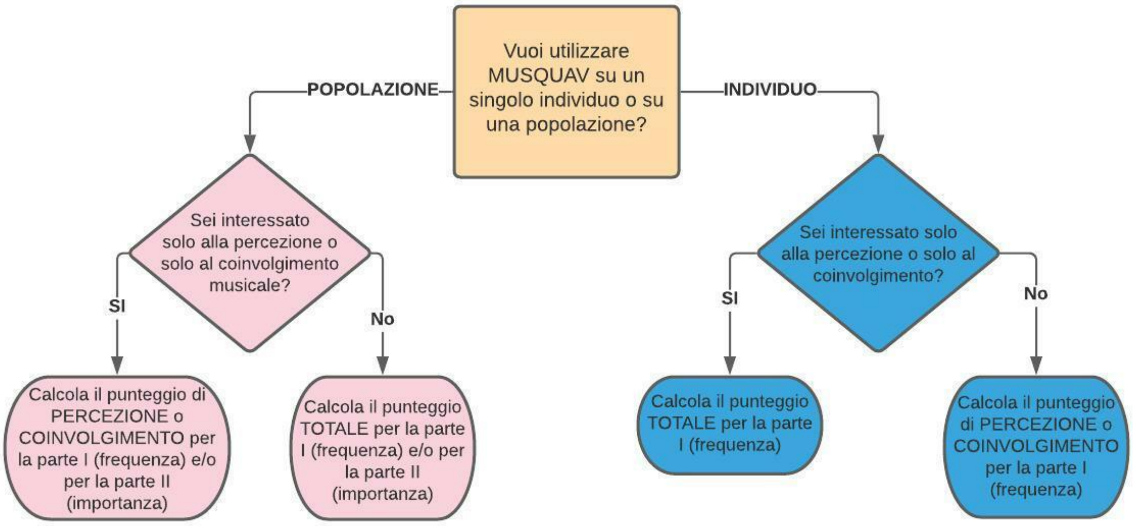

Bibliografia:

- Dritsakis G, van Besouw RM, Kitterick P, Verschuur CA. A Music-Related Quality of Life Measure to Guide Music Rehabilitation for Adult Cochlear Implant Users. Am J Audiol. 2017 Sep 18;26(3):268-282. doi: 10.1044/2017_AJA-16-0120. PMID: 28614845.

- Frosolini A, Parrino D, Mancuso A, Coppola N, Genovese E, de Filippis C. THE MUSIC-RELATED QUALITY OF LIFE: ITALIAN VALIDATION OF MURQOL INTO MUSQUAV QUESTIONNAIRE AND PRELIMINARY DATA FROM A COHORT OF POSTLINGUALLY DEAFENED COCHLEAR IMPLANT USERS. European Archives of Oto-Rhino-Laryngology and Head & Neck (2022). https://doi.org/10.1007/s00405-022-07258-1
